# Isolation, cultivation and immunostaining of single myofibers: An improved approach to study the behavior of satellite cells

**DOI:** 10.14440/jbm.2018.219

**Published:** 2018-03-16

**Authors:** Chai Ling Lim, King-Hwa Ling, Pike-See Cheah

**Affiliations:** 1 Department of Human Anatomy, Faculty of Medicine and Health Sciences, Universiti Putra Malaysia, 43400 UPM Serdang, Selangor, Malaysia; 2 Department of Biomedical Sciences, Faculty of Medicine and Health Sciences, Universiti Putra Malaysia, 43400 UPM Serdang, Selangor, Malaysia; 3 Genetics and Regenerative Medicine Research Centre, Faculty of Medicine and Health Sciences, Universiti Putra Malaysia, 43400 UPM Serdang, Selangor, Malaysia

**Keywords:** adult stem cell, myofibers, myogenic differentiation 1, paired box family of transcription factors, satellite cell

## Abstract

Satellite cells are myogenic cells responsible for muscle growth shortly after birth and muscle repair/regeneration during adulthood. Therapies based on satellite cells hold promise for treating muscular dysfunctions. Studying satellite cells is technically challenging owing to their low abundance, small size and anatomical dispersed location between the basal lamina and the sarcolemma of myofibers. In this article, we present three improved protocol strategies for studying the properties of satellite cells of the mouse during the different stages of muscle regeneration: (1) immunostaining of freshly isolated single myofibers to facilitate the study of quiescent satellite cells, (2) cultivation of single myofibers on Matrigel^®^-coated dish to study the myogenesis programs initiated by satellite cell activation, and (3) cultivation of single myofibers in floating conditions to analyze activated satellite cells or the doubling time of satellite cells in myofibers. In brief, when compared to previously published protocols, this article presented an improved protocol that requires shorter experimental time and less laborious approach for higher yield of intact single myofibers for downstream analyses.

## BACKGROUND

Skeletal muscle is a highly complex and heterogeneous tissue that plays essential roles in voluntary body movements, insulating internal organs, and producing heat to maintain core body temperature, as well as acting as a major energy storage depot. Human have approximately 639 to 640 skeletal muscle units, which together account for around 38% and 30% of total body mass in men and women, respectively [1].

Skeletal muscle has a robust and effective regenerative capacity. Muscle function can be restored even after the tissue is removed, minced and replaced *in situ* [2]. This regenerative capacity is mainly attributed to a population of cells in skeletal muscle, called satellite cells. Satellite cells (also known as skeletal muscle stem cells) are rare mononuclear cells that have a low cytoplasmic content and are wedged between the basal lamina and the sarcolemma of postnatal skeletal muscle. In adult skeletal muscle, satellite cells are mitoticaly quiescent under normal circumstances, but they can be activated in response to exercise and trauma [3]. Activated satellite cells will migrate to the site of injury, proliferate and differentiate to generate new myofibers [4]. Since satellite cells possess such an outstanding capacity in muscle regeneration, they have garnered great attention from scientists.

Studying satellite cells can be technically challenging. Their scarcity, small size, dispersed location under the basal lamina of myofibers and their non-random distribution along the length of individual myofibers makes them difficult to be identified and manipulated [3,4]. Additionally, it is laborious to separate the satellite cells from other cells present within skeletal muscle (*e.g.*, fibroblasts, endothelial cells, interstitial cells, and blood vessel-associated cells), in order to obtain a pure population of satellite cells. These factors have limited the study of satellite cells. Therefore, an approach known as single myofiber isolation aims to isolate satellite cells while retaining them within their niches was established by Dr. Richard Bischoff and his colleagues in 1975 [5]. They demonstrated that myofibers physically peeled from adult rat muscle and juvenile quail muscle could be cultured to study muscle regeneration events [5,6]. Their protocol was subsequently modified by Rosenblatt and colleagues to allow the isolation and culture of longer and more fragile myofibers derived from mouse extensor digitorum longus (EDL), soleus and tibialis anterior (TA) muscles [7]. Since then several modifications have been proposed which aim to improve the yields and the handling of the isolated myofibers [8-11].

Methods for single myofibers isolation provide essential tools for studying the biological properties of satellite cells during the different stages of myogenesis. It allows studying satellite cells in their *in situ* position as well as examining their progeny after migrating from the myofibers. For examples, the freshly isolated myofibers can be either immunostained immediately to characterize the quiescent satellite cells [12] or cultured in serum-rich growth medium for studying the interplay between the parent myofiber and its associated satellite cells. When cultured in suspension form, isolated myofibers can be used for analysis of cell division of individual satellite cells. Additionally, this method also enables the enrichment of pure satellite cells or cells of a myogenic lineage *in vitro* [7].

Previously published protocols incorporate more complex and time-consuming procedures to isolate single myofibers. It is essential that the isolation duration to be performed as short as possible. The prolonged isolation time will induce the activation of satellite cells and eventually leading to the inaccuracy of the results [13]. It is common that the survivability of the myofibers decreases during the digestion and trituration procedure. The number of myofibers will also reduce during the myofibers picking and immunostaining procedure. Hence, it is of the utmost importance that the isolation protocol will lead to a great yield of intact myofibers in a single preparation. Thus, we report an improved and rapid protocol for the isolation, cultivation, and immunostaining of viable myofibers derived from the EDL muscles of adult mice. We have detailed the procedure and illustration to harvest the EDL muscle by using the tendon-to-tendon approach. We also highlight crucial steps and revised strategies to facilitate better yield of healthy single myofibers. In addition, we introduced a step-by-step approach for effective immunostaining of the freshly isolated myofiber-associated satellite cells for studying the quiescent satellite cells. Taken together, we believe that this improved protocol will be beneficial for researchers interested in studying satellite cells.

## MATERIALS

### Reagents

- Collagenase type 1 (Sigma-Aldrich^®^, St. Louis, MO, USA; cat. #C0130)
- Matrigel^®^ in growth factor reduced format (Corning^®^ Inc, Corning, NY, USA; cat. #354230)
- Dulbecco’s Modified Eagle Medium with 4500 mg/l L-glucose, 4.0 mM L-glutamine, and 110 mg/l sodium pyruvate (DMEM) (Gibco^®^, Paisley, Scotland, UK; cat. #11995-073)
- Standard and non-heat inactivated horse serum (HS) (HyClone Laboratories, Inc, Logan, UT, USA; cat. #SH30074.03)
- Standard and non-heat inactivated fetal bovine serum (FBS) (Gibco^®^, Paisley, Scotland, UK; cat. #10270-106)
- Chicken embryo extract powder (Gemini Bio-products, West Sacramento, CA, USA; cat. #100-163P)
- Penicillin-Streptomycin with 10000 units/ml penicillin and 10000 μg/ml streptomycin (Gibco^®^, Paisley, Scotland, UK; cat. #15140-122)
- Crystal phosphate buffer saline (PBS) (Bioline Reagents Ltd, London, UK; cat. #BIO37108)
- Crystalline paraformaldehyde (PFA) powder (Sigma-Aldrich^®^, St. Louis, MO, USA; cat. #P6148)
- Triton X-100 reagent (Sigma-Aldrich^®^, St. Louis, MO, USA; cat. #T8787)
- Tween-20 reagent (Sigma-Aldrich^®^, St. Louis, MO, USA; cat. #P9416)
- Goat serum (Gibco^®^, Paisley, Scotland, UK; cat. #16210-064)
- Mouse monoclonal anti-Pax7 (Developmental Studies Hybridoma Bank, Iowa City, Iowa, USA; clone: Pax7)
- Mouse monoclonal anti-myogenic differentiation 1 (MyoD) (clone: 5.8am; Thermo Fisher Scientific Inc, Waltham, Massachusetts, USA; cat. #MA5-12902)
- Goat anti-mouse Alexa Fluoro 488 Ig G1 secondary antibody (Invitrogen, Carlsbad, CA, USA; cat. #A21121)
- ProLong^®^ Gold Antifade reagent containing 4, 6-diamidino-2-phenylindole (DAPI) (Invitrogen, Carlsbad, CA, USA; cat. #p36935)
- SuperFrost™ Plus microscope slides (Menzel-Glaser Laboratories, Braunschweig, Germany; cat. #J1800AMNZ)
- Liquid repellent PAP pen (Cosmo Bio, Koto-ku,Tokyo, Japan; cat. #DAI-PAP-S-M)

### Recipes

#### 0.2% (w/v) of collagenase type 1 solution (6 ml)

Dissolve 12 mg of collagenase type 1 powder in 6 ml of DMEM. Filter-sterilize the mixture using 0.22 μm syringe filter.

#### Washing media (50 ml)

Supplement 49.5 ml of DMEM with 500 μl of penicillin-streptomycin.

**CAUTION:** It is recommended to use DMEM with sodium pyruvate throughout all of the procedure to ensure myofiber survival.

#### Chicken embryonic extract solution (10 ml)

Dissolve 10 g of chicken embryonic extract powder in 10 ml of 1× PBS solution. Filter-sterilize the mixture using 0.22 μm syringe filter.

#### HS–containing culture media (50 ml)

Supplement 44.5 ml of DMEM with 5 ml of HS (10% v/v) and 500 μl of penicillin-streptomycin.

#### Myofiber culture media (50 ml)

Supplement 34 ml of DMEM with 10 ml of FBS (20% v/v), 5 ml of HS (10% v/v), 500 μl of chicken embryo extract (1% v/v) and 500 μl of penicillin-streptomycin.

#### 10% (v/v) Matrigel^®^ working solution (10 ml)

Dilute 1 ml of Matrigel^®^ stock solution with 9 ml of ice-cold DMEM.

#### 4% (w/v) of paraformaldehyde (100 ml)

Dissolve 4 g of crystalline PFA powder with 100 ml of sterilized water and subsequently incubate the mixture in a 55°C water bath until the PFA powder is fully dissolved. Adjust the pH value to pH 7.4 with sodium hydroxide. Filter-sterilize the mixture using a 0.22 μm filtration unit.

**NOTES:** The 4% PFA solution must be prepared in a fume hood.

#### 0.5% (v/v) of Triton X-100 detergent (50 ml)

Supplement 49.75 ml of 1× PBS with 250 μl of Triton X-100 reagents.

#### 1% (v/v) of blocking buffer (10 ml)

Supplement 9.9 ml of 1× PBS with 100 μl of goat serum.

#### 0.05% (v/v) Tween-20 washing solution (50 ml)

Supplement 49.975 ml of 1× PBS with 25 μl of Tween-20.

#### Mouse monoclonal anti-Pax7 working solution (400 μl)

Supplement 320 μl of 1% (v/v) of blocking buffer with 80 μl of mouse monoclonal anti-Pax7 stock solution.

#### Mouse monoclonal anti-MyoD working solution (400 μl)

Supplement 398 μl of 1% (v/v) of blocking buffer with 2 μl of mouse monoclonal anti-MyoD stock solution.

#### Alexa Fluoro 488 Ig G1 secondary antibody working solution (1000 μl)

Supplement 999 μl of 1% (v/v) of blocking buffer with 1 μl of Alexa Fluoro 488 Ig G1 secondary antibody stock solution.

### Equipment

- Standard humidified CO_2_ incubator (37°C, 5% CO_2_ supply) (RS Biotech Laboratory Equipment Ltd, Irvine, Scotland, UK; cat. #Mini Galaxy^®^ A)
- Tissue culture hood (Clyde-Apac, NSW, Australia; cat. #BHA90)
- Diamond pen (VWR Scientific Inc, Radnor, Pennsylvania, USA; cat. #52865-005)
- Standard 9-inch glass Pasteur pipettes (VWR Scientific Inc, Radnor, Pennsylvania, USA; cat. #0035904)
- Bunsen burner (Sigma-Aldrich^®^, St. Louis, MO, USA; cat. #Z270318)
- Rubber pipette bulbs
- Dissecting board
- Dressing forceps (stainless steel, rounded points with serrated tips and 140 mm length) (VWR Scientific Inc, Radnor, Pennsylvania, USA; cat. #25601-072)
- Micro-dissecting forceps (stainless steel, very fine curved points and 110 mm length) (VWR Scientific Inc, Radnor, Pennsylvania, USA; cat. #25607-856)
- Micro-dissecting scissors (stainless steel, sharp point and 101.6 mm) (Sigma-Aldrich^®^, St. Louis, MO, USA, cat. #S3146)
- 0.22 μm PVDF low protein binding syringe filters (Millex-GS, Merck Millipore, Billerica, Massachusetts, USA; cat. #SLGV033RS)
- 3 cc disposable plastic syringes (Becton Dickinson, Franklin Lakes, NJ, USA, cat. #309657)
- 60 mm^2^ plastic culture dishes (TPP Techno Plastic Products AG, Zollstrasse, Trasadingen, Switzerland; cat. #93060)
- 1.5 ml round-bottom microcentrifuge tubes (Axygen Scientific, Union City, CA, USA; cat. #MCT-150-C)
- 24-well plastic tissue culture dishes (TPP Techno Plastic Products AG, Zollstrasse, Trasadingen, Switzerland; cat. #92024)
- Stereo microscope (Nikon, Minato, Tokyo, Japan; cat. #SMZ745T)
- Fluorescence microscope (Olympus, Shinjuku-ku, Tokyo, Japan; cat. # Olympus BX51 fluorescence microscope equipped with an UplanF1 objective lens and Olympus ANALYSIS^®^ FIVE acquisition software)
- Inverted microscope (Nikon, Minato, Tokyo, Japan; cat. #Nikon Eclipse TS100/100-F microscope equipped with Nikon DS-Fil and Nikon NIS-Elements D Imaging Software)

## PROCEDURE

**Figure 1.**
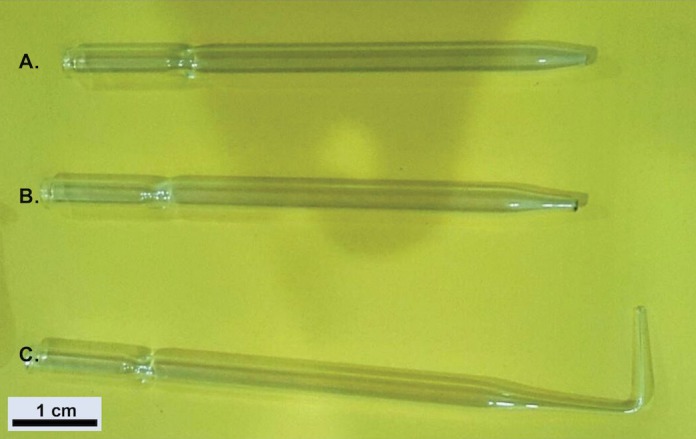
A set of glass Pasteur pipettes with different bore diameter. **A.** The widest bore pipette. **B.** A narrower bore pipette. **C.** The finest bore pipette with a small aperture.

**Figure 2.**
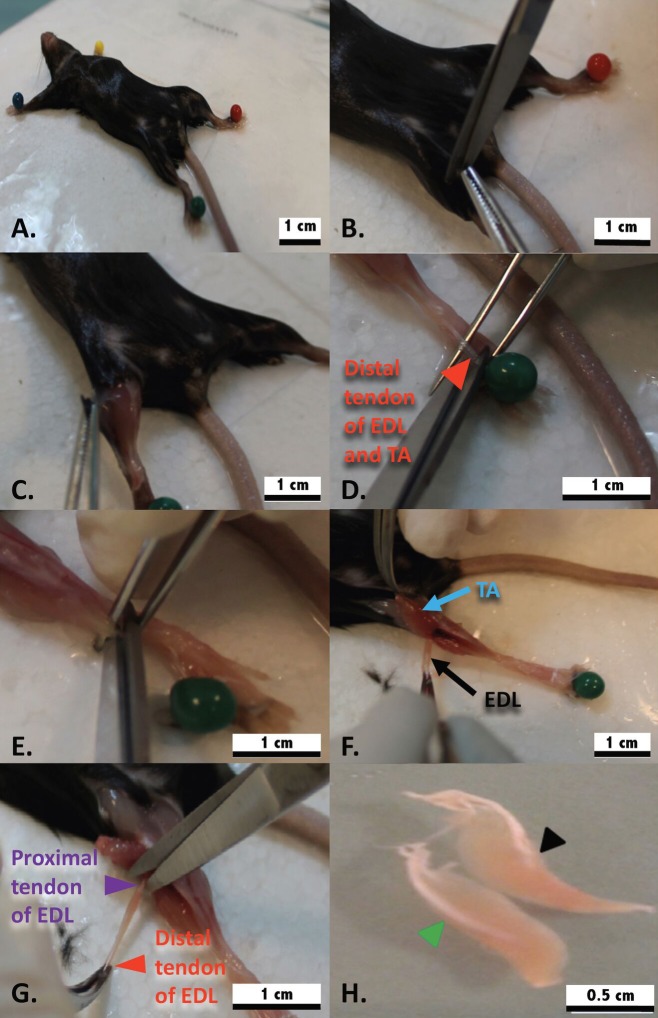
Tendon to tendon of the adult C57BL/6 mouse aged postnatal (P) 56 d. **A.** The mouse was secured in supine position on the dissecting board. **B.** The skin from above the knee to the paw was incised. **C.** The skin was retracted to expose the musculature. **D.** The insertion points of the distal tendons of EDL and TA (red arrowhead) were identified. **E.** The distal tendons of EDL and TA were excised. **F.** EDL (black arrow) and TA (blue arrow) were separated by clamping at their respective tendons. **G.** The tendon of EDL was cut very closely at its proximal end (purple arrowhead). **H.** A comparison between the intact EDL (black arrowhead) and damaged EDL (green arrow head). The intact EDL is fusiform in shape with tapering ends while the damaged EDL appears as a tissue chunk with blunt distal ends.

**Figure 3.**
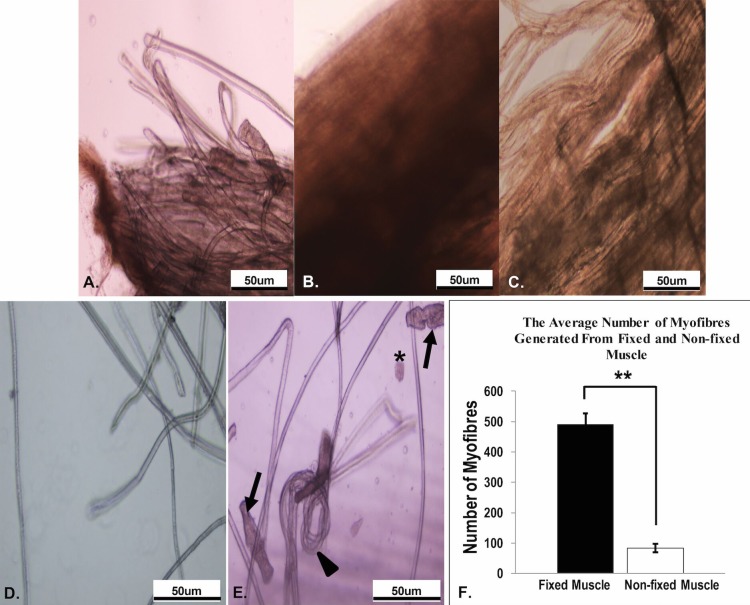
Representative microscopy images that show the conditions during enzymatic digestion and liberation of single myofibers. **A.** A well-digested EDL looks less defined and slightly swollen with many hairlike single myofibers loosened from the edge. **B.** The under-digested EDL looks more compact with the myofibers are tightly bundled together. **C.** The over-digested EDL looks slack and it would break into pieces during sample processing. **D.** Higher yield of healthy and intact myofiber isolated from the fixed muscle. **E.** Hypercontracted myofibers (arrows), distorted myofibers (arrowheads) and debris (asterisk) are often seen during liberation from the non-fixed muscle. **F.** The average number of myofibers generated from fixed and non-fixed myofibers. The data as shown as the mean ± SEM of three biological replicates (*n* = 3). Statistical significance was calculated by using a Student’s *t*-test. The average number of myofibers generated from the fixed muscle was statistically significant (*P* = 0.00307) compared to the non-fixed EDL muscle. ^**^P < 0.01.

1. Preparation prior to muscle harvesting
  **1.1.** Pasteur pipettes of different sizes for single myofibers isolation
    A set of Pasteur pipettes with bore diameters ranging from 1 mm to 3 mm is needed for different stages of the myofiber isolation procedure (**Fig. 1**). The bore pipette with the widest diameter is used to transfer digested muscle from dish to dish (**Fig. 1A**). The bore pipette with a narrower diameter is used to triturate digested muscle in order to release single myofibers (**Fig. 1B**). The finest bore Pasteur pipette is used to isolate healthy myofibers (**Fig. 1C**). The procedures for the generation of Pasteur pipettes set are as detailed below:
    **1.1.1.** Cut the glass Pasteur pipette using a diamond pen and snap away the cutting end to create a set of pipettes with different bore diameter sizes.
    **1.1.2.** Flame polish the sharp ends in the hottest part of a Bunsen flame (non-luminous flame near the tip of the inner blue cone) until the sharp edges are softened and smoothened.
    **1.1.3.** Autoclave the flame-polished glass Pasteur pipettes.
    **1.1.4.** Coat the pipettes with HS by passing undiluted filtered HS through them several times.
  **1.2.** HS-coated 1.5 ml round-bottom microcentrifuge tube for the immunocytochemistry staining of the freshly isolated myofibers.
    **1.2.1.** Transfer 1 ml of HS per 1.5 ml round-bottom microcentrifuge tube and swirl the tube gently to ensure even coating.
    **1.2.2.** Allow the tubes to sit for 5 min at room temperature.
    **1.2.3.** Discard HS and air dry the tubes for at least 30 min at room temperature.
  **1.3.** HS-coated 60 mm^2^ plastic Petri dishes for single myofibers culture
    **1.3.1.** Transfer 1 ml of HS per 60 mm^2^ plastic Petri dish and then swirl the dishes gently to ensure even coverage with serum.
    **1.3.2.** Incubate the dish for 5 min at room temperature.
    **1.3.3.** Discard the HS and allow the dishes to air-dry for at least 30 min at room temperature.
    **1.3.4.** Transfer 6 ml of washing media into each dish. Keep the dishes with washing medium in the CO_2_ incubator prior to use.
      **HINTS:** HS-coated plastic and glasswares should be used throughout the isolation procedure to prevent adhesion of single myofibers to the walls of those plastic and glasswares prior to plating or immunocytochemistry staining. Alternatively, FBS can be used as a coating agent.
  **1.4.** Matrigel^®^-coated 24-well culture dishes for myofiber culture.
    **1.4.1.** Pipette 500 μl of 10% (v/v) Matrigel^®^ solution into each well of ice-chilled 24-well dishes.
    **1.4.2.** Gently swirl the 24-well dishes to ensure an even coating with Matrigel^®^.
    **1.4.3.** Allow the dishes to sit for 20 min at 4°C.
    **1.4.4.** Discard the Matrigel^®^ solution. Incubate the dishes in the CO_2_ incubator for at least 30 min prior to use.
      **HINTS:** Matrigel^®^ will solidify at temperatures > 10°C. Therefore, throughout the coating procedure always keep the Matrigel^®^ solution chilled. The plastic pipette tips, conical tubes and 24-well dishes should also be pre-chilled to prevent premature gelling.
2. Animal handling and euthanasia
  All experiments involving animal breeding and handling were approved by the Universiti Putra Malaysia Institutional Animal Care and Use Committee (IACUC) and were performed in accordance with institutional regulations on experimental animals (Reference number: UPM/IACUC/AUP-R003/2014). All animals involved in this study were adult male C57BL/6 mice at postnatal (P) days 90 and they were bred under controlled environmental conditions of a 12 h light/dark cycle at 21°C–23°C with a relative humidity of 55%. A standard pellet diet and water were available *ad libitum*. All mice were euthanized by cervical dislocation after being anesthetized in a chamber containing 2.5% isoflurane in 100% oxygen.
  **NOTES:** In this experiment, all mouse involved were adult male C57BL/6 mice at postnatal (P) days 90. The enzymatic digestive conditions (collagenase concentration and incubation time) stated in this, step 4 serves as a reference only. The experimental conditions are optimal for the EDL muscle derived from the mouse with the same background. The researcher may optimize the enzymatic digestive conditions by taking into consideration on the age, gender, size and the strain of the mouse. The outlooks of a well-digested EDL and the important points for the enzymatic digestive procedures are detailed in step 4.
3. Harvest the EDL using a tendon to tendon approach
  **3.1.** Sterilize all working area surfaces and dissection tools with 70% ethanol.
  **3.2.** Sterilize the entire mouse body with 70% ethanol and place the mouse in a supine position on the dissection board. Subsequently, secure all limbs to the dissection board using big head pins (**Fig. 2A**).
  **3.3.** Make a small incision in the skin at the anterior knee area. Extend the incision vertically towards the paws (**Fig. 2B**). Retract the skin lateral to expose underlying musculoskeletal structures (**Fig. 2C**).
  **3.4.** Identify the distal TA and EDL tendons under a stereomicroscope and cut them using sharp micro-dissecting scissors (**Fig. 2D**-**2E**).
  **3.5.** Grip the distal TA and EDL tendons with curved tip micro-dissecting forceps and gently lift them upwards. The EDL is located underneath the TA. Separate the EDL from the TA by pulling the individual tendons in opposite directions (**Fig. 2F**).
  **3.6.** Cut the proximal end of the EDL tendon and transfer the tissue into a Petri dish containing 6 ml of washing media (washing dish) (**Fig. 2G**).
    **CRITICAL STEP:** It is important to isolate the muscle from tendon to tendon in order to maintain myofiber integrity. A well dissected/undamaged EDL should be long and slender (**Fig. 2H**).
4. Enzymatic digestion
  **4.1.** Transfer the EDL to a Petri dish containing 6 ml of 0.2 % (w/v) collagenase type 1 solution.
  **4.2.** Incubate the muscle inside the humidified CO_2_ incubator for 90 min. Gently swirl the dish every 10–15 min during the digestion. The muscle should be examined regularly under a microscope to avoid over-digestion that may lead to myofiber hyper-contraction.
    **CRITICAL STEP:** Collagenase digestion is a critical step in determining the success of the single myofibers isolation procedure. The collagenase concentration and incubation time should be adjusted based on the age, gender, size and the strain of the mouse. Longer digestion times will be required for male, larger and/or more aged muscle.
    **HINTS:** A well-digested EDL looks less defined and slightly swollen, and has many hairlike single myofibers loosened from the edge (**Fig. 3A**). Single myofibers can be detached from the muscle with gentle shaking.
  **4.3.** When digestion is complete, use the widest bore Pasteur pipette to transfer the digested EDL to another Petri dish containing washing media.
  **4.4.** Transfer the digested EDL to a second, third and then fourth Petri dish containing washing medium to further dilute any residual collagenase and wash away any remaining debris.
  **4.5.** The digested EDL can be used for different downstream analyses (1) immediately fixed, triturate and immunostaining for Paired box family of transcription factors (Pax7) to study quiescent satellite cells (step 5.1), (2) triturated and cultured on a substrate such as Matrigel^®^-coated dish to investigate *in vitro* myogenesis events (step 5.2) and (3) triturated and cultured in floating conditions for at least 48 h before fixation and immunostaining for MyoD to investigate the activated satellite cells or doubling time of satellite cells in myofibers (step 5.3).
5. Downstream analyses
  **5.1.** Liberation and immunocytochemistry staining of freshly isolated single myofibers—To investigate quiescent satellite cells
    **5.1.1.** After enzymatic digestion and several times of washing, carefully transfer the digested EDL bundle into a HS-coated 1.5 ml round-bottom microcentrifuge tube using the widest bore glass Pasteur pipette. Subsequently, fixed the digested EDL bundle with 800 μl of 4% (w/v) PFA solution for 10 min.
    **5.1.2.** After 10 min of fixation, carefully remove 4% (w/v) PFA solution and gently rinse the EDL bundle with 1 ml of 1× PBS (3× for 10 min each) to remove residual PFA.
    **5.1.3.** Transfer the PFA-fixed EDL bundle into a 60 mm^2^ plastic Petri dish and subsequently triturate the muscle bundle gently with the widest bore glass Pasteur pipette to release individual myofibers. After 5 min of trituration, an uncountable number of single myofibers would be liberated from the muscle bulk.
      **HINTS:** The diameter of the muscles will decrease as time goes. Consequently, repeat the titration process with a narrower bore Pasteur pipette until the required amount of myofiber was obtained.
    **5.1.4.** Next, with the aid of a stereomicroscope, select and transfer the straight and non-fragmented myofibers into a HS-coated 1.5 ml rounded bottom microcentrifuge tube using a HS-coated Pasteur pipette with a small aperture in the middle.
    **5.1.5.** Place the tube in an upright position to allow the myofibers to sink to the bottom of the tube. Later, carefully remove the 1× PBS from the tubes using the finest bore Pasteur pipette inserted just below the liquid surface. Maintain the 1× PBS to a height of 5 mm above the myofibers.
      **CAUTION:** Ensure that the myofibers sink to the bottom of microcentrifuge tubes before aspirating and changing the reagents during the immunostaining procedure. A sufficient amount of solution (5 mm above the level of the myofibers) is needed to prevent the loss of myofibers during the immunostaining procedure.
    **5.1.6.** Permeabilize the myofibers with 800 μl of 0.5% (v/v) Triton X-100 detergent for 8 min at room temperature. Then, discard the detergent and gently rinse the myofibers with 1 ml of 1× PBS (3× for 5 min each) before immuno-blocking with 800 μl of 1% (v/v) blocking buffer for at least overnight at 4°C with gentle agitation (*e.g.*, on a shaker).
    **5.1.7.** Remove the blocking solution and subsequently incubates the myofibers with 200 μl of the primary antibody (pre-diluted to 1:5 in 1% (v/v) blocking buffer) for overnight at 4°C with gentle agitation (*e.g.*, on a shaker).
    **5.1.8.** On the next day, remove the antibody and rinse the myofibers with 1 ml of 0.05% (v/v) Tween-20 washing solution (3× for 5 min each). Next, incubate the myofibers for 1 h in a dark room with 200 μl of Alexa Fluoro 488 Ig G1 secondary antibody (pre-diluted to 1:1000 in 1% (v/v) blocking buffer).
    **5.1.9.** Discard the secondary antibodies and rinse the myofibers with 1 ml of 0.05% (v/v) Tween-20 washing solution (3× for 5 min each).
    **5.1.10.** Use a PAP pen to outline a liquid repelling barrier on the SuperFrost^TM^ Plus microscope slide.
      **NOTES:** The use of SuperFrost™ Plus microscope slides is strongly recommended to prevent myofiber detachment.
    **5.1.11.** Add 500 μl of 1× PBS into microcentrifuge tubes containing immunostained myofibers and gently pour the mixture into a Petri dish pre-coated with horse serum. Subsequently, rinse the microcentrifuge tubes with another 500 μl of 1× PBS to remove remaining myofibers.
    **5.1.12.** Collect myofibers under a stereomicroscope and transfer them onto the SuperFrost™ Plus microscope slide with a drop of ProLong^®^ Gold Antifade Reagent with DAPI. Finally, cover the slide gently with a glass cover slip.
      **NOTES:** This transfer step should be performed as quickly as possible to prevent photo-bleaching.
  **5.2.** Liberation and culture of single myofibers—To study myogenesis programs initiated by satellite cell activation
    **5.2.1.** Prepare seven Petri dishes containing washing media and label as “1^st^ trituration dish”, “2^nd^ trituration dish”, “collection dish”, “washing dish 1”, “washing dish 2”, “washing dish 3” and “incubation dish”. Pre-warm these dishes in a CO_2_ incubator at 37°C.
    **5.2.2.** Transfer the digested EDL bundle into the 1^st^ trituration dish and place the dish in the CO_2_ incubator for 10 min.
    **5.2.3.** Gently triturate the digested EDL bundle with the widest bore glass Pasteur pipette to release individual myofibers. After 5 min of trituration, approximately 30 viable single myofibers will be liberated from the digested EDL.
    **5.2.4.** Transfer the remaining EDL bundle from the 1^st^ trituration dish into the 2^nd^ trituration dish. Repeat the trituration process with a narrower bore Pasteur pipette. Meanwhile, place the 1^st^ trituration dish containing viable myofibers in the CO_2_ incubator to keep the myofibers warm.
      **CAUTION:** It is crucial to maintain the media containing the myofibers at 37°C as the myofibers will fragment if the temperature of the medium drops below 37°C for an extended period of time.
    **5.2.5.** Repeat the EDL transferring and trituration process as described above until the desired number of myofibers are collected.
      **NOTES:** Do not over force the single myofibers to be released from the muscle bulk. Excessive force used during trituration will lead to the damage and the fragmentation of myofibers. If the myofibers remain tightly bundled together, the researcher shall digest the muscle bulk again in the freshly prepared 0.2% (w/v) collagenase type 1 solution for another 10–15 min. The morphology of the digested muscle should be examined regularly under a microscope to avoid over digestion.
    **5.2.6.** Next, with the aid of a stereomicroscope, carefully select only straight, healthy myofibers and reject partially contracted myofibers.
    **5.2.7.** Transfer the selected myofibers from the trituration dish to the collection dish using the finest bore Pasteur pipette with a small aperture.
    **5.2.8.** Wash the myofibers at least three times in washing medium by transferring them from the collective dish to the first, second, third washing dish and then incubation dish. This step will remove all dead/short hyper-contracted myofibers and other debris.
    **5.2.9.** Incubate those myofibers in a CO_2_ incubator at 37°C in the incubation dish for at least 1 h prior to switching to a culture medium. This allows myofibers to adjust to the *in vitro* conditions in the absence of serum.
      **CAUTION:** An immediate culture of myofibers in serum rich medium will increase myofibers shrinking.
    **5.2.10.** After at least one hour of incubation, transfer a Matrigel^®^-coated 24-well dish from the CO_2_ incubator to a laminar-flow hood. Add 250 μl of pre-warmed myofiber culture media into each well of the Matrigel^®^-coated 24-well dishes.
    **5.2.11.** Transfer individual myofiber, taking care to avoid transferring residual medium, and gently release the myofiber in the center of the well.
      **HINTS:** Refresh the HS-coated Pasteur pipette by passing undiluted filtered HS through them several times before transferring the myofibers to prevent myofibers from adhering to the pipette wall.
    **5.2.12.** Culture the myofibers in the 24-well dishes in a CO_2_ incubator at 37°C.
      **NOTES:** Avoid moving the dish for at least overnight as the myofibers are loosely adhere to the bottom of the well.
    **5.2.13.** After three days, add 250 μl of culture medium to the myofibers. After three subsequent days, replace the old medium with 500 μl of fresh culture medium.
      **NOTES:** Chicken embryonic extract contains numerous components that have stimulatory and/or inhibitory effects on the cell cycle of satellite cells [14]. Therefore, the use of non-ultra purified/ non-centrifuged chicken embryonic extract is highly recommended.
  **5.3.** Culture of single myofibers in floating conditions—To investigate activated satellite cells or doubling time of satellite cells in myofibers
    **5.3.1.** Prepare a 60 mm^2^ plastic culture dish containing 5 ml pre-warmed HS containing culture medium. Pre-warm that dish in a CO_2_ incubator at 37°C.
    **5.3.2.** Transfer the selected myofibers from the incubation dish to the culture dish using the finest bore Pasteur pipette with a small aperture (Proceed from step 5.2.8). Culture the myofibers in a CO_2_ incubator at 37°C for 48 h or the longer period prior to immunocytochemistry staining for MyoD.
    **5.3.3.** After 48 h or longer period in culture, transfer those myofibers into a HS-coated 1.5 ml rounded bottom microcentrifuge tube using a HS-coated Pasteur pipette with a small aperture in the middle.
    **5.3.4.** Immunostain the myofibers with MyoD (pre-diluted to 1:200 in 1% (v/v) blocking buffer).

## ANTICIPATED RESULTS

Satellite cells play a distinct role in the routine skeletal muscle maintenance and repair of damaged adult skeletal muscles. A small defect in this population of cells can lead to the delayed, impaired or failure of muscle regeneration responses. Here, we present a reliable and simple technique for the isolation, cultivation, and immunostaining of the viable myofibers to study the characteristics of satellite cells during different stages of the myogenesis. Results and details on the critical point of each step are illustrated below:

### Dissect the EDL muscle from tendon to tendon to maintain the integrity of muscle

In this study, the EDL muscle was harvested *via* the tendon to tendon approach in order to maintain the integrity of muscle. Muscle integrity is essential to determine the success rate of the procedure. An intact muscle facilitates better yield of the intact myofibers while the damaged muscle will lead to the poor yield and fragmented myofibers. **Figure 2H** is the representative image that shows the appearance of an intact (black arrowhead) and a damaged (green arrowhead) EDL muscle. The intact EDL is fusiform in shape while the damaged EDL appears as tissue chunk with a blunt distal end.

### Optimal enzymatic digestion conditions are crucial to obtaining well-digested skeletal muscle

Collagenase digestion is another critical step that determines the success of the single myofibers liberation procedure. The under- or over-digestion of muscle tissue can lead to a poor yield of intact myofibers. In this experiment, the EDL muscle was digested with 0.2% (w/v) collagenase type 1 solution at 37°C for 90 min. The collagenase digestion conditions, such as collagenase concentration and incubation time used here were optimized based on the conditions of EDL isolated from adult C57BL/6 male mouse aged postnatal (P) day 90. Ideally, the enzymatic digestion conditions should be adjusted based on the age, size, gender and the strain of the mouse. For example, longer digestion time is required for larger and/or more aged muscle. Therefore, it is highly recommended to monitor the digestion of muscle regularly under a microscope to avoid over-digestion that may lead to myofiber hyper-contraction. **Figure 3** are the representative microscopy images that show the morphology of an EDL muscle in three digestive stages, well-digested, under-digested and over-digested. A well-digested EDL looks less defined and slightly swollen and has many hairlike single myofibers loosened from the edge (**Fig. 3A**). The under-digested EDL appeared compacted and the myofibers are still tightly bundled together (**Fig. 3B**). The over-digested EDL looked slack and will be fragmented easily during handling (**Fig. 3C**).

### Liberation of myofibers after fixation preserves the satellite cells as close to the quiescent state as possible

Myofibers are delicate and satellite cells tend to be activated during the trituration procedures. In the published protocol, single myofibers are liberated from the digested muscle bulk and the liberation condition should be maintained at 37°C to prevent the fragmentation of myofibers [12,15]. Hence, the muscle sample is required to be transferred back and forth to the CO_2_ incubator to re-equilibrate the liberation temperature. The complexity, tediousness and prolonged isolation procedure will induce activation of the satellite cells. Gnocchi and colleagues suggested that the myofibers should be fixed within 2 h after animal euthanization to preserve the associated satellite cells as near to the quiescent state as possible [13]. In this refined protocol, the well-digested muscle bulk was immediately fixed for 10 min in 4% (w/v) PFA after the enzymatic digestion procedure. When compared to previously published protocols [8,11,12], this procedure reduces the complexity of procedure and shorten the time of the experiment by 2–3 h between the step of the end of enzymatic digestion and the step of the beginning of the fixation; subsequently, it preserves the associated satellite cells as close to the quiescent state as possible (**Table 1**). In addition, the refined protocol also generated a significantly higher yield of intact and viable myofibers (*P* = 0.003) as compared to the non-fixed muscle (**Fig. 3D** and **3E**). Approximately 400–500 intact myofibers were liberated from the fixed EDL muscle, whereas only 50–100 intact myofibers were harvested from the non-fixed EDL muscle (**Table 1** and **Fig. 3F**).

### Immunostaining of the satellite cells with Pax7 and MyoD markers

**Figure 4.**
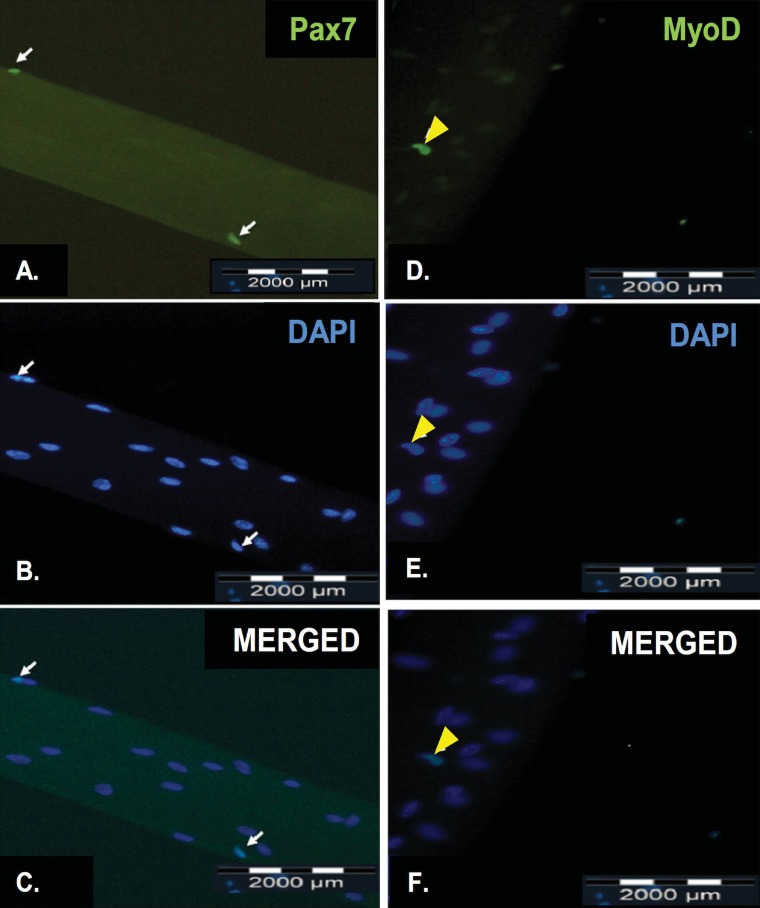
Representative results of immunofluorescence staining of satellite cells on a freshly isolated myofiber and on a myofiber after cultured in floating condition for 72 h. Pax7 is expressed in the nuclei of quiescent satellite cells (A, white arrow) while MyoD is expressed in the nuclei of activated satellite cells (D, yellow arrowhead). All myonuclei present were revealed by counterstaining with DAPI (B and E). The merged fluorescence image of both Pax7-staining and DAPI-staining of a quiescent satellite cell (C, white arrow). The merged fluorescence image of both MyoD-staining and DAPI-staining of an activated satellite cell (F, yellow arrow). The scale bar represents 2000 μm.

Using the refined protocol, we have identified both quiescent and activated satellite cells using Pax7 and MyoD, respectively. Pax7 and MyoD were expressed in the nuclei of quiescent (white arrow) and activated (yellow arrowhead) satellite cells, respectively. (**Fig. 4A** and **4D**). All myonuclei present were revealed by counterstaining with DAPI (blue) (**Fig. 4B** and **4E**).

### Additional step of collagenase digestion during the titration procedure improves the yield and quality of intact myofibers

Liberation of single myofibers by trituration using Pasteurs pipette is one of the most critical procedure that determines the yields and quality of myofibers. The inadequate and excessive force used during trituration will lead to poor yields of intact myofibers. This procedure can be technically challenging, especially when handling the muscle with greater mass owing to the lack of exposure of the innermost layer of muscle tissue to the collagenase solution. To overcome this problem, the repetition of enzymatic digestion should be included, when necessary. This strategy allows the collagenase enzyme to further digest the connective tissues between each myofibers in the innermost layer of tissue and, thereby, reduce the compactness of muscle bulk. Consequently, the single myofibers can easily released from the muscle bulk during trituration. Using the improved protocol, we can yield an approximately 200–300 intact myofibers, whereas the published protocols can produce 50–100 intact myofibers from the same mass of muscle tissue (**Table 2**).

### Cultivation of isolated single myofibers

**Figure 5.**
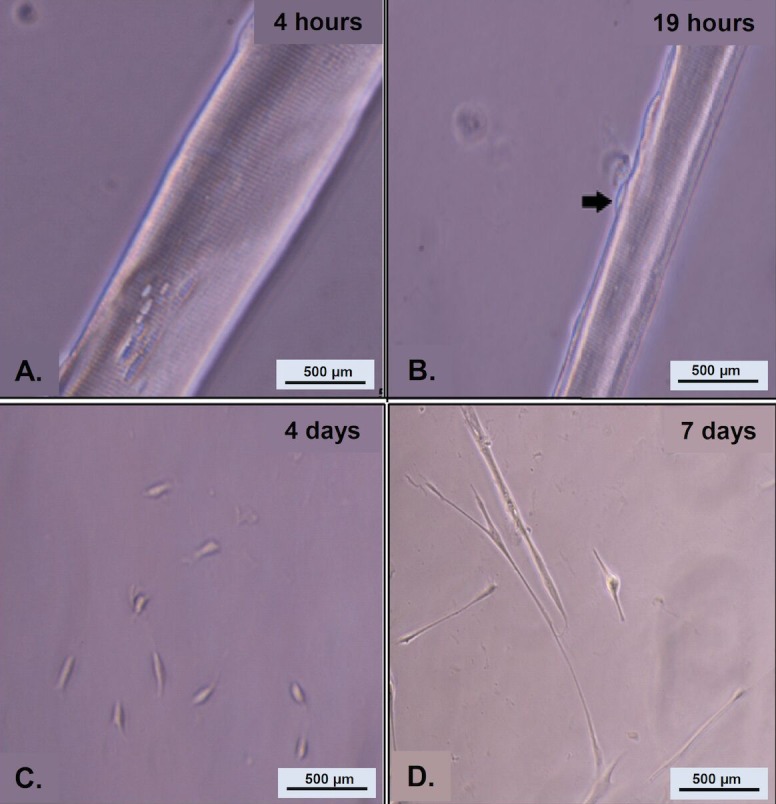
Phase micrographs of EDL myofibers depicting the temporal development of myogenic cultures of cells emanating from individual myofibers at 4 h (A), 19 h (B), 4 d (C) and one week (D).

To study myogenesis program initiated by stellite cell activation, single myofibers were isolated and cultured individually in 24-well dishes coated with Matrigel^®^ for a week. Cultures were maintained in serum-rich medium, as described in this protocol. **Figure 5A** shows that the satellite cells remained attached to the myofibers four hours after culturing. After 19 h in culture, the satellite cells begin to migrate away from the myofiber (**Fig. 5B**). Satellite cells that migrated from the myofibers continued to proliferate, differentiate, and fuse into myotubes (**Fig. 5C** and **5D**).

## Troubleshooting

Possible problems and their troubleshooting solutions are listed in **Table 3**.

## Figures and Tables

**Table 1.** Comparisons of the refined protocol and the previously published protocols for the isolation and immunocytochemistry staining of freshly isolated single myofibers.

Criteria	Refined protocol	Published protocols [8-12]
Tediousness	Simple	Laborious
Total time required	1/3 shorter	
Yields	Higher (400 to 500 intact myofibers)	Lower (50 to 100 intact myofibers)

**Table 2.** Comparisons of the refined protocol and the previously published protocols for the isolation and cultivation of single myofibers.

Criteria	Refined protocol	Published protocols [7-9,11]
Yields	Higher (200 to 300 live myofibers)	Lower (50 to 100 live myofibers)
Simplicity to release single myofibers during trituration	Easier	More difficult
Survivability of myofibers during isolation	Better	Lower
Quality of the isolated myofibers	More isolated myofibers presented with straight, intact and non fragmented morphology	More isolated myofibers are bent and fragmented

**Table 3.** Troubleshooting.

Step	Problems	Probable causes	Suggestions
5.2.3.	Myofiber release during trituration is sub-optimal	Muscle is under-digested	Increase the collagenase incubation time
5.2.5.	Low yield of intact myofibers; high numbers of hypercontracted myofibers and high amounts of debris	Muscle was damaged Muscle was over-digested	Dissect muscle from tendon-to-tendon Decrease the collagenase incubation time
5.2.12.	Myofibers do not survive after myofiber plating	The selected myofibers were not healthy and were partially contracted	Select only straight and healthy myofibers and avoid transferring partially contracted myofibers
5.2.12.	Fibroblasts are growing together with myofibers in the culture dish	The selected myofibers are contaminated with debris An inadequate number of washes	Selected debris-free myofibers Increase the number of washes

## References

[1] Kyle UG Dupertuis YM Raguso CA Hans D Pichard C (2003) Appendicular skeletal muscle mass (ASMM) percentiles in 7489 healthy adults aged 18–98 years determined by bioelectrical impedance analysis (BIA). Clin Nutr 22: 1-10. 14613755 10.1016/s0261-5614(03)00048-7

[2] Studitsky AN (1964) FREE AUTO- AND HOMOGRAFTS OF MUSCLE TISSUE IN EXPERIMENTS ON ANIMALS. Ann N Y Acad Sci 120: 789-801. doi: 10.1111/j.1749-6632.1964.tb34771.x. PMID: 14235292

[3] Boldrin L Morgan JE (2012) Human satellite cells: identification on human muscle fibres. PLoS Curr 3: 1294-10. doi: 10.1371/currents.RRN1294. PMID: 22333991 PMC3275414

[4] Yin H Price F Rudnicki MA (2013) Satellite cells and the muscle stem cell niche. Physiol Rev 93: 23-67. doi: 10.1152/physrev.00043.2011. PMID: 23303905 PMC4073943

[5] Bischoff R (1975) Regeneration of single skeletal muscle fibers in vitro. Anat Rec 182: 215-235. doi: 10.1002/ar.1091820207. PMID: 168794

[6] Konigsberg UR Lipton BH Konigsberg IR (1975) The regenerative response of single mature muscle fibers isolated in vitro. Dev Biol 45: 260-275. doi: 10.1016/0012-1606(75)90065-2. PMID: 1193298

[7] Rosenblatt JD Lunt AI Parry DJ Partridge TA (1995) Culturing satellite cells from living single muscle fiber explants. In Vitro Cell Dev Biol Anim 31: 773-779. doi: 10.1007/BF02634119. PMID: 8564066

[8] Shefer G Yablonka-Reuveni Z (2005) Isolation and culture of skeletal muscle myofibers as a means to analyze satellite cells. Methods Mol Biol 290: 281-304. PMID: 15361669 10.1385/1-59259-838-2:281 PMC3523695

[9] Anderson JE Wozniak AC Mizunoya W (2012) Single muscle-fiber isolation and culture for cellular, molecular, pharmacological, and evolutionary studies. Methods Mol Biol 798: 85-102. doi: 10.1007/978-1-61779-343-1_6. PMID: 22130833

[10] Verma M Asakura A (2011) Efficient single muscle fiber isolation from alcohol-fixed adult muscle following β-galactosidase staining for satellite cell detection. J Histochem Cytochem 59: 60-67. doi: 10.1369/jhc.2010.956730. PMID: 20876523 PMC3201122

[11] Pasut A Jones AE Rudnicki MA (2013) Isolation and culture of individual myofibers and their satellite cells from adult skeletal muscle. J Vis Exp 22: 50074-10. doi: 10.3791/50074. PMID: 23542587 PMC3639710

[12] Beauchamp JR Heslop L Yu DS Tajbakhsh S Kelly RG (2000) Expression of CD34 and Myf5 defines the majority of quiescent adult skeletal muscle satellite cells. J Cell Biol 151: 1221-1234. doi: 10.1083/jcb.151.6.1221. PMID: 11121437 PMC2190588

[13] Gnocchi VF White RB Ono Y Ellis JA Zammit PS (2009) Further characterisation of the molecular signature of quiescent and activated mouse muscle satellite cells. PLoS One 4: doi: 10.1371/journal.pone.0005205. PMID: 19370151 PMC2666265

[14] Gharaibeh B Lu A Tebbets J Zheng B Feduska J (2008) Isolation of a slowly adhering cell fraction containing stem cells from murine skeletal muscle by the preplate technique. Nat Protoc 3: 1501-1509. doi: 10.1038/nprot.2008.142. PMID: 18772878

[15] Fukada S Uezumi A Ikemoto M Masuda S Segawa M (2007) Molecular signature of quiescent satellite cells in adult skeletal muscle. Stem Cells 25: 2448-2459. doi: 10.1634/stemcells.2007-0019. PMID: 17600112

